# Place of Death of Cancer Patients Treated at a German Comprehensive Cancer Center

**DOI:** 10.1089/pmr.2024.0097

**Published:** 2025-05-22

**Authors:** Julia Berendt, Maria Heckel, Christoph Ostgathe, Stephanie Stiel, Peter Stachura, Andreas Becker, Matthias W. Beckmann, Susanne Gahr

**Affiliations:** ^1^ Department of Palliative Medicine, University Hospital Erlangen, Friedrich-Alexander-Universität Erlangen-Nürnberg (FAU), Comprehensive Cancer Centres, CCC Erlangen-EMN, Comprehensive Cancer Centres Alliance WERA (CCC WERA), Bavarian Cancer Research Centres (BZKF), Erlangen, Germany.; ^2^ Institute for General Practice and Palliative Care, Hannover Medical School, Hanover, Germany.; ^3^ Department of Anesthesiology and General Intensive Care, University Hospital St. Poelten, St. Poelten, Austria.; ^4^ Medical Informatics and Communication Centres, University Hospital Erlangen, Friedrich-Alexander-Universität Erlangen-Nürnberg (FAU), Comprehensive Cancer Centres, CCC Erlangen-EMN, Comprehensive Cancer Centres Alliance WERA (CCC WERA), Bavarian Cancer Research Centres (BZKF), Erlangen, Germany.; ^5^ Department of Gynecology and Obstetrics, University Hospital Erlangen, Friedrich-Alexander-Universität Erlangen-Nürnberg (FAU), Comprehensive Cancer Centres, CCC Erlangen-EMN, Comprehensive Cancer Centres Alliance WERA (CCC WERA), Bavarian Cancer Research Centres (BZKF), Erlangen, Germany.

**Keywords:** cancer, end-of-life care, home care, hospital, palliative care, place of death

## Abstract

**Background:**

Public health research includes end-of-life care. Place of death is an indicator of end-of-life care quality.

**Objective:**

We assessed the place of death of cancer patients treated at a Comprehensive Cancer Center (CCC), caring for an average of 2220 primary cases per year.

**Methods:**

Dataset includes information on cancer patients who were treated at least once in a German CCC, died between 2009 and 2013, and for whom a place of death could be assigned. Data—reported following the “REporting of Studies Conducted Using Observational Routinely Collected Data” guideline—were retrieved from death registration and analyzed retrospectively. Descriptive analyses, frequency calculations, Pearson/Cramer’s V chi-square tests, and *t* tests in SPSS 28.0 were used.

**Results:**

A total of 5855 patients were analyzed (metastases *n* = 2830, 48.3%; recurrent cancer *n* = 1930, 33.1%). Finally, 3523 (60.2%) died in a clinical setting (CCC: 28.9%/other hospital: 31.3%). Patients who died in the CCC (mean age 66.3 years) were younger than those who died in other hospitals (mean age 67.8 years; *p* = 0.034) or at home (ø 70.2 years; *p* = 0.000). Cancer patients who died in the CCC (*n* = 1693) had over time a median of 356 contacts with specialized palliative care within 30 days before death (standard deviation [SD]: 319–377, mean 352). One-third of patients died within one year of diagnosis (*p* < 0.001). For patients dying in the CCC, the rate was even higher (50.6%, *p* < 0.001).

**Conclusion:**

Even if treated in certified centers, CCC cancer patients have a high in-hospital mortality rate. The place of death reflects care structures and disease progression, highlighting the need for palliative care. As frequent death sites, CCCs should offer specialized palliative services. Further research is needed to better align the place of death with patient wishes.

## Introduction

In the contemporary era of patient autonomy,^1^ individuals have extensive opportunities to govern their own health care and where and when to die. People did not recognize the end of life (EOL) as a significant public health issue for a long time. However, discussing individual preferences is part of a person-centered approach in health care.^2,3^ Advance care planning and shared decision making include discussions about the person’s preferred place for EOL care.

Internationally, place and preferred place of deaths (POD) are acknowledged as quality indicators for EOL care,^4–6^ especially for palliative care services.^7,8^ Involving the immediate family and social environment in care planning is equally important.^9,10^ Most patients prefer to die in familiar settings,^11^ but many eventually spend their last days and hours of life in hospitals. The desired and actual POD seldom align.^11^ The percentage of deaths in hospitals varies,^12–14^ with the highest numbers in South Korea (85%) and lower rates, for example, in the Netherlands with about 25%.^15–17^ However, in Germany (46%)^13,18^ and England (47%),^3,16^ many people die in the hospital. In Germany, the ratio between hospital and private deaths correlates with gender.^19^ Here, more male cancer patients die in hospitals than female cancer patients. Trends in Europe exhibit considerable regional and economic disparities, characterized by low and declining rates in the northwest and high and increasing prevalence in the south and east.^14^ At the same time, palliative care has been increasingly accessed,^20^ and providing palliative care has led to a decrease in hospital deaths.^14^

Treatment in certified centers, compared with noncertified ones, is linked to a lower mortality rate and longer overall survival for patients diagnosed with incidental cancer. In recent decades, the percentage of cancer patients treated at certified centers in Germany has increased.^21^

German Cancer Aid’s “Oncological Centers of Excellence” initiative has fostered the establishment of Comprehensive Cancer Centers (CCCs), the third level of certification, at several university medical centers over the past decade. CCCs are top-notch urban medical facilities that specialize in treating complex, challenging cases. They are particularly well suited to modern cancer treatment.^22^ Europe’s Beating Cancer Plan aims to establish a European Network that links recognized National Comprehensive Cancer Centers in every Member State by 2025.^23^ In Germany, of almost 500,000 cancer patients diagnosed annually, 90,000 are treated in CCCs, and as many as 240,000 contact the CCCs just for a second opinion.^24^ CCCs distinguish themselves from other facilities by applying exceptional knowledge and equipment in interdisciplinary teams to treat uncommon and extremely complex diseases.^25^

Therefore, CCCs in Germany are only funded if they provide a palliative care service.^26^ CCCs serve as a regular, local provider of care and may be or have an impact on the POD for cancer patients.

To explore the role of a regional CCC on the POD, this study addresses the following primary research questions: Where do patients treated in a CCC die? Do certain patient characteristics influence the POD? Second, it was explored on the basis of the available data: How frequently was specialized palliative care integrated within 30 days before death at the CCC?

## Methods

### Study design

The “REporting of Studies Conducted Using Observational Routinely Collected Data” guideline guided the planning of the study, which involved a retrospective observatory analysis of routinely collected health data.^27^ We performed data reconstruction and analysis in Erlangen to study trends on a smaller scale, confirming the feasibility of implementing data pooling and analysis for the entire CCC network in Germany. The study received approval from the local ethics committee (22_15 Bc).

### Data source

Retrospective analysis linked electronic data from three databases: the Regional Cancer Registry from Bavaria, Germany; the SAP® System; and the Registry Office for Civil Data in Erlangen. In the period under review from 2010 till 2013, the Comprehensive Cancer Center Erlangen-European Metropolitan Region of Nuremberg (EMN) treated on average 2220 primary cases per year (median 2037).

### Data extraction and collection

A retrospective, anonymized data collection was initiated. Data collection from the electronic death registration system took place from July to November 2016 in the Erlangen registry office. Collaborators signed the appropriate data protection declarations and then listed the required information on an Excel sheet to ensure that patient data were anonymized.

### Study population

Data include information on all cancer patients (i) who were treated at least once in the CCC Erlangen, (ii) who died between 2009 and 2013, and (iii) for whom a POD could be assigned.

Cancer deaths are identified through ICD-10 codes C00–C97 (malignant neoplasm). Five diagnosis groups (C40–C41, C45–C49, C73–C75, C76–C80, and C44) were excluded because they were too small in sample size and/or nonspecific.

### Data analysis

**FIG. 1.**
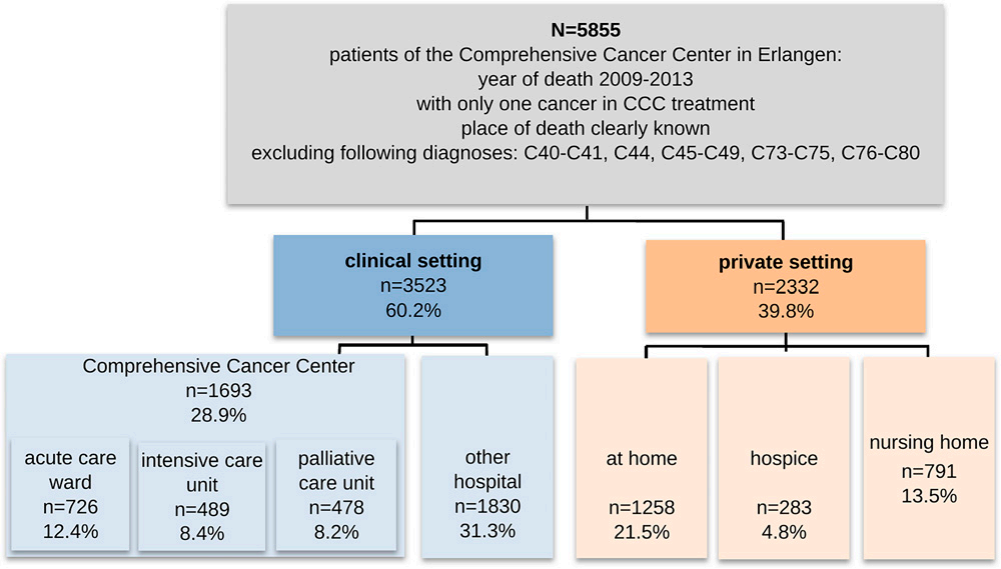
Distribution of place of death.

The analysis includes a descriptive analysis of the places of death. We dichotomized the groups into clinical and private settings (Fig. 1). The POD at the CCC was further subdivided into palliative care unit, acute care ward, and intensive care unit (differentiated POD). In Germany, hospices are usually not part of the hospital; in fact, they are independent home-like institutions when acute care on a palliative care unit is no longer neccessary.^28^ The patient data were analyzed using SPSS 28.0 (frequencies, percentage, and significance).

In addition, this population-based study analyzed differences in POD regarding gender, age, existence of metastases, diagnosis, time of death, and time in relation to the establishment of the palliative care unit of the CCC (University Hospital) in Erlangen. If the mean values of two independent samples were different, a *t* test for continuous variables was conducted. We performed further comparisons using the chi-square test for trend. A two-sided *p* ≤ 0.05 was considered statistically significant. Cramer’s V as an effect size measurement for the chi-square test of independence was also analyzed.

In addition, we performed binomial logistic regression, meaning we specified the groups and checked to determine groups based on the dependent variable, for example, clinical and private setting.

## Results

### Data selection/composition of data

Of the 9394 patients who were diagnosed with a cancer disease recorded in the Cancer Registry Erlangen-Nuremberg and died between 2009 and 2013, 79.2% (*n* = 7438) had a POD that was clearly allocatable. We analyzed 5855 patients after eliminating those with multiple cancer diagnoses and those with small sample sizes. Characteristics are shown in Table 1.

**Table 1.** Characteristic of Study Population (*N* = 5855)

Characteristics		Number (*n*)	Percent (%)
Gender			
Male		3230	55.2
Female		2625	44.8
Age of death in years (median age 69 ± SD 14)			
0–17		20	0.3
18–34		75	1.3
35–64		1872	32.0
65–74		1691	28.9
75–84		1487	25.4
≥85		710	12.1
Primary cancer prior to first therapy			
Yes		545	9.3
No		5310	90.7
Recurrent cancer			
Yes		1930	33.1
No		3917	66.9
Metastasis			
Yes		2830	48.3
No		3025	51.7
Malignant neoplasms			
Solid tumors		5355	91.5
Hematological malignancies		500	8.5
Primary diagnosis			
C00–C14	Malignant neoplasms of lip, oral cavity, and pharynx	490	8.4
C15–C26	Malignant neoplasms of digestive organs	1826	31.2
C30–C39	Malignant neoplasms of respiratory and intrathoracic organs	637	10.9
C43	Malignant melanoma of skin	306	5.2
C50	Malignant neoplasm of breast	641	10.9
C51–C58	Malignant neoplasms of female genital organs	373	6.4
C60–C63	Malignant neoplasms of male genital organs	357	6.1
C64–C68	Malignant neoplasms of urinary tract	367	6.3
C69–C72	Malignant neoplasms of eye, brain, and other parts of central nervous system	358	6.1
C81–C96	Malignant neoplasms of lymphoid, hematopoietic, and related tissue	500	8.5
Duration from diagnosis until death in months			
0–12		1946	33.2
>12		3909	66.8
Contact with specialized palliative care unit within 30 days before death			
No		5202	88.8
Yes		653	11.2

SD, standard deviation.

### Distribution regarding place and setting for dying

Of all deaths, 60.2% (*n* = 3523) took place in a clinical setting and 39.8% (*n* = 2332) in a setting that was more private (at a home, in hospice, or in a nursing home). The CCC was POD for 28.9% of the deceased; 8.2% (*n* = 478) of 1693 CCC patients died on the palliative care unit (8.2% of the whole sample, *n* = 5855). Within the private settings, the majority of patient deaths occurred at home (*n* = 1258, 21.5%) (Fig. 1).

### POD and age

Patients who passed away in a CCC (mean age 66.3 years) were significantly younger than those who passed away in other hospitals (mean age 67.8 years; *p* = 0.034) or at home (mean age 70.2 years; *p* = 0.000). According to the recommendations of Backhaus et al. from 2021^29^, the regression model had a poor variance resolution (no effect) of Nagelkerke’s *R*^2^ = 0.04 and was statistically significant (x^2^(5) = 195.63, *p* = 0.001). With a sensitivity of 23.5% and a specificity of 91.2%, the overall percentage of correctly classified samples was 95.1%. The likelihood of passing away in a private setting rose with age by a factor of 1.027. The probability of dying in a private setting, i.e., outside a hospital, increases by 2.7% with each additional year.

### POD and cancer diagnoses

The statistical correlation between diagnosis and differentiated POD is weak (Cramer’s V = 0.1; *p* < 0.001). Among all patients, those with malignant neoplasms of hematopoietic and lymphoid tissues (76.0%; *p* = 0.000), respiratory and intrathoracic organs (64.4%, *p* = 0.012), and the urinary tract (54.2%, *p* = 0.010) were significantly more likely to die in a clinical setting. In addition, patients with central nervous system cancer were significantly more likely to die in a private setting (57.0%; *p* = 0.000) (Table 2).

**Table 2.** Place of Death and Cancer Diagnosis (*N* = 5855)

Primary diagnosis	ICD-10	Place of death clinical setting	Place of death private setting
Malignant neoplasms of lip, oral cavity, and pharynx	C00–C14	304 (62.0%)	186 (38.0%)
Malignant neoplasms of digestive organs	C15–C26	1119 (61.3%)	707 (38.7%)
Malignant neoplasms of respiratory and intrathoracic organs	C30–C39	410 (64.4%)	227 (35.6%)^*^
Malignant melanoma of skin	C43	188 (61.4%)	118 (38.6%)
Malignant neoplasm of breast	C50	368 (57.4%)	273 (42.6%)^*^
Malignant neoplasms of female genital organs	C51–C58	232 (62.2%)	141 (37.8%)
Malignant neoplasms of male genital organs	C60–C63	169 (47.3%)	188 (52.7%)^*^
Malignant neoplasms of urinary tract	C64–C68	199 (54.2%)	168 (45.8%)^*^
Malignant neoplasms of eye, brain, and other parts of central nervous system	C69–C72	154 (43.0%)	204 (57.0%)^*^
Malignant neoplasms, stated or presumed to be primary, of lymphoid, hematopoietic and related tissue	C81–C96	380 (76.0%)	120 (24.0%)^*^
Overall		3523 (60.2%)	2332 (39.8%)

^*^ *p* < 0.05.

### POD and metastases

Of all cases that died in the clinical setting (*N* = 3523), 48.9% had metastases (*n* = 2830), while 47.4% of patients (*n* = 1106) died in the private setting (*p* = 0.135). However, there is a weak statistical correlation between the presence of metastases and the more differentiated places of death (Cramer’s V = 0.2; *p* < 0.001). In detail, 56.6% passed away in an acute hospital ward of the CCC, regardless of the palliative care unit and intensive care unit. Seventy-two percent of patients who passed in the palliative care unit had metastases; only 19% of patients with metastases passed in the intensive care unit. Sixty percent of patients without metastases and 40% of patients with metastases pass away in nursing homes, respectively. In hospice, 40% of patients without metastases and 60% of patients with metastases pass away.

### POD and gender

Of the patients who passed away in a clinical setting (*n* = 3523), males made up a higher proportion (57.1%, *n* = 2013) than females (42.9%; *n* = 1217; *p* = 0.000). However, male patients with genital organ malignant neoplasm (*n* = 357) were more likely to experience death in a private setting (52.7%; *n* = 188; *p* = 0.000; see Table 2). In general, there is a weak correlation between gender and the differentiated POD (Cramer’s V = 0.1; *p* < 0.001).

### Contact palliative care within 30 days before death

At the CCC, there were no structures for specialized palliative care until 2009. Prior to this, palliative care could not be financed using the corresponding ICD-10 codes. After the establishment of the palliative medicine department in 2010, the percentage of patients who died in the CCC (*n* = 1693) with contact to specialized palliative care within 30 days before death has increased from the early days of the newly established palliative medicine department with 21.6% to 43.0% death per year in the CCC (SD: 319–377; mean 352; median 356).

### POD and duration from diagnosis to death

Overall, 33.2% (*n* = 1946) of all patients (*n* = 5855) died within the first year of receiving a cancer diagnosis. For patients dying in the CCC, this was even higher (50.6%; *n* = 367; *p* < 0.001) (Table 3).

**Table 3.** Place of Death and Duration from Diagnosis to Death in Months

Place of death	In total	Duration from diagnosis to death ≤12 months	Duration from diagnosis to death >12 months
**Clinical setting**	**3523 (100%)**	**1293 (36.7%)**	2230 (63.3%)^*^
CCC, University Hospital Erlangen^a^	726 (100%)	367 (50.6%)	359 (49.4%)^*^
Palliative care unit	478 (100%)	229 (47.9%)	249 (52.1%)^*^
Intensive care unit	489 (100%)	231 (47.2%)	258 (52.8%)^*^
Other hospital	1830 (100%)	466 (25.5%)	1364 (74.5%)^*^
**Private setting**	**2332 (100%)**	**653 (28.0%)**	1679 (72.0%)^*^
Home	1258 (100%)	330 (26.2%)	928 (73.8%)^*^
Hospice	283 (100%)	92 (32.5%)	191 (67.5%)
Care facility	791 (100%)	231 (29.2%)	560 (70.8%)^*^
Overall	5855 (100%)	1946 (100%)	3909 (100%)

The significance of both values in bold is *p* < .001.

^*^ *p* < 0.05.

^a^ Without palliative care and intensive care unit.

CCC, Comprehensive Cancer Center.

## Discussion

The CCC, as it is part of the local provision of care, is not only a place for diagnosis, treatment, and research but also an important place of care and POD. The data presented here are one of several recent studies in Germany focusing on patients treated at a CCC, but other studies were published in other regions,^12,13,30^ and neither provided a detailed classification of the POD nor considered places of death in a palliative care setting.

We found that 60.2% of CCC patients died in hospitals, which is at the upper level of the range previously published for cancer patients and their POD in Germany spanning from 49.0% to 62.8%.^13,18,31,32^ The rather high number may be explained by the complexity of provisions provided within CCCs.^33^ This is underpinned by the finding that patients with metastases—probably seeking new treatment options in the CCC—were more likely to die in a clinical setting. In addition, CCCs in Germany have broad catchment areas for referrals from other medical facilities and for second opinions, due to their interdisciplinary setup and novel diagnostic and treatment options.

However, recent data show that, in general, over the last decade, fewer patients die in hospitals, with more patients choosing palliative care and hospice for EOL care.^13^ Nevertheless, the CCC Erlangen EMN, Germany, and other countries^15^ seem to exceed quality benchmarks (<17% of cancer patients should die in hospital^4^), which, however, were not specifically developed for CCC patients with rather complex needs. This benchmark could be adapted to be a more realistic indicator for good oncology and palliative care in a CCC. Because requirements concerning excellent EOL care in hospitals remain one of the most important aspects of highly advanced care supply, education for integrating palliative care teams and incorporating palliative care knowledge into therapy and treatment is invaluable for supporting cancer patients and their loved ones. The intensified integration of palliative care into hospital care can obviously have an impact on the number of deaths in hospitals.^14^

Age has a small but significant influence on the probability of dying at home, as shown by a low-power model. It seems that the risk of dying in a private setting, i.e., outside a hospital, increases by 2.7% with each additional year. However, patients who died in the hospital were more frequently male,^18^ younger than the average cancer patient who died elsewhere, and diagnosed with malignant neoplasms of hemopoietic and lymphoid tissues, intrathoracic organs, or the urinary tract. At the same time, we know that male patients—as underlined in a study by Sharma et al.—more frequently express a wish for aggressive therapy and more often reject palliative care.^34^ As Saeed’s study demonstrates, women appear to be more open to palliative care than men.^35^ Therefore, public health strategies should be developed and implemented to better inform male patients about palliative care options available in a CCC and its referral institutions. The number of CCC patients dying in a clinical setting with more than 12 months after diagnosis, including deaths in the palliative care unit, in the intensive care unit, or in other hospitals, far exceeds the number of patients with less than 12 months. To date, no studies have shown a relation between the time since cancer diagnosis and the POD. Taking a closer look at the individual locations of the clinical setting category, over half of the patients (50.6%) who die within 12 months after diagnosis do so at CCC itself. However, this did not include patients in the intensive care unit, the palliative care unit, and all other hospitals. Available studies which report without a time limit after diagnosis indicate that, above all, patients with nonsolid cancers like leukemia or lymphoma have an increased risk of dying in hospital.^19,36^ Nonsolid cancers may be treated using a curative therapy approach until the very end, which could explain this phenomenon.^37^

The fact that 40% of patients died in a private setting is consistent with other studies showing that strong social and family support increases the probability of dying at home at the EOL.^38,39^ As stated earlier, most people wish to die at home.^36,40^ Advanced care planning and the creation of advance directives would be useful with this in mind, yet these aspects have so far been neglected.^41^

## Conclusion

Of all CCC patients who died within the CCC, a significant proportion of 28.2% spent their last days of life on the CCC’s palliative care unit. Specialized palliative care has been increasingly integrated into CCC Erlangen and has become a more favorable option in cases of incurable disease for EOL care. However, the majority still die on the oncological ward, showing an urgent need to strengthen generalist palliative care with easy access to specialist services (e.g., palliative care hospital support teams) throughout the whole CCC, even if basic training and application of palliative care skills are available. The proportion of patients who rather wish to die in a hospital must always be taken into account.

A CCC, in particular, offers broader and partially more aggressive curative cancer treatment options^42^ for patients with higher disease complexity, which also increases the likelihood of a patient passing away in the hospital.^43^ Therefore, the CCC needs awareness of and information about palliative care among staff from different medical disciplines as well as patients and informal carers to offer and foster counseling and treatment services, including inpatient and outpatient specialized palliative care services. It should therefore also be in the interest of the CCC leadership to track places of death in order to respond with appropriate care structures within a CCC and in the region of a CCC to ensure good EOL care for their patients. The data could help to better understand disease progression in oncology and palliative care, improve care structures and interfaces, and increase awareness of cooperation in the outpatient sector.

## Limitations

We manually merged data from various sites, including the cancer registry, the CCC, and the registry office. Comprehensive socioeconomic data were not used as a basis. The manual documentation effort required to compare the POD’s address with the address list of surrounding institutions was potentially error-prone. Only the data of one CCC were examined. The ECOG Perfortmance Status was not filtered out from the hospital information system. The reason for this is as follows: The ECOG Status is not recorded regularly in the hospital and is handled differently in the hospital departments, so that the significance of the ECOG Status did not seem obvious. In addition, there would be no comparability between the groups because the ECOG Status in the hospice and at home before/with death is generally not recorded. There was no recording of the preferred POD for individual EOL patients. No judgments on the quality of EOL care could be made.

## Abbreviations

CCC: Comprehensive Cancer Center
ECOG: Eastern Cooperative Oncology Group
EMN: European Metropolitan Region of Nuremberg
EOL: end of life
ICD: International Classification of Diseases
POD: place of death
SD: standard deviation
SPSS: Statistical Package für Social Sciences
